# Predictive Contribution of p16 Beyond HPV Genotype in the Histopathologic Detection of CIN2+ Lesions: A Real-World Cervical Biopsy Cohort Study

**DOI:** 10.3390/diagnostics16172784

**Published:** 2026-08-29

**Authors:** Eda Güner Özen, Süleyman Özen, Duygu Tanyeri, Uygar Tanyeri, Muzaffer Sancı

**Affiliations:** 1 Department of Obstetrics and Gynecology, İzmir City Hospital, İzmir 35040, Türkiye; 2 Department of Gynecologic Oncology, İzmir City Hospital, İzmir 35040, Türkiye; 3 Department of Pathology, İzmir City Hospital, İzmir 35040, Türkiye

**Keywords:** cervical intraepithelial neoplasia, CIN2+, human papillomavirus, HPV genotype, p16INK4a, Ki-67, cervical biopsy, risk stratification, immunohistochemistry, predictive modeling

## Abstract

**Background**: HPV genotype-based stratification estimates cervical disease risk but does not directly measure cellular transformation. We assessed whether p16 and Ki-67 improve prediction of CIN2+ beyond HPV genotype and age in a biopsy-confirmed referral cohort. **Methods**: This retrospective study included 531 high-risk HPV-positive women evaluated between December 2023 and January 2026 who had either margin-negative excisional histopathology or completed 12-month surveillance after conservative management. HPV genotypes were grouped as HPV16, HPV18, or other high-risk types. Independent associations were examined using prespecified multivariable logistic models; discrimination, calibration, 2000-resample bootstrap validation, and a uniform index-biopsy-reference sensitivity analysis were evaluated. **Results**: CIN2+ was confirmed in 191/531 women (36.0%). HPV16 positivity (adjusted odds ratio [aOR] 1.72, 95% confidence interval [CI] 1.15–2.57), age (aOR 1.027 per year, 95% CI 1.011–1.043), and p16 positivity (aOR 2.97, 95% CI 1.81–4.89) independently predicted CIN2+, whereas Ki-67 did not (aOR 1.74, 95% CI 0.90–3.35; *p* = 0.098). Adding p16 to age and genotype increased the AUC from 0.608 to 0.704 (ΔAUC 0.097, bootstrap 95% CI 0.057–0.138); Ki-67 increased it only to 0.707 (ΔAUC 0.003, 95% CI −0.002 to 0.015). Optimism-corrected AUCs were 0.599, 0.696, and 0.697, respectively. The uniform biopsy-reference analysis produced the same pattern. **Conclusions**: p16 provides reproducible incremental risk information beyond age and HPV genotype in biopsy-confirmed cervical disease, whereas Ki-67 adds little once p16 is known. Neither biomarker should replace histopathology-guided management.

## 1. Introduction

Cervical cancer remains a substantial global health challenge despite effective primary and secondary prevention strategies. In 2020, an estimated 604,127 new cases and 341,831 deaths occurred worldwide, with approximately 90% of deaths concentrated in low- and middle-income countries, reflecting persistent inequities in HPV vaccination, screening, and timely treatment [1]. Persistent infection with oncogenic human papillomavirus (HPV) is the necessary cause of cervical carcinogenesis; however, most infections clear spontaneously, and only a minority progress to high-grade intraepithelial disease or invasive cancer [2,3].

Oncogenic potential varies substantially across HPV genotypes. HPV16 has the highest carcinogenicity and persistence, followed by HPV18 and several other high-risk types, including HPV31, 33, 45, 52, and 58 [4]. Genotype-specific risk has therefore been incorporated into contemporary screening and management algorithms [5]. Nevertheless, genotype is a virologic classification rather than a direct marker of cellular transformation. Its discriminative capacity may be attenuated in referral cohorts in which colposcopy-directed biopsy has already enriched the prevalence of disease.

Histopathologic assessment remains central to management after abnormal cervical screening. Interpretation is nevertheless variable, particularly for CIN2, which occupies an intermediate and biologically heterogeneous position between productive and transforming HPV infection [6]. This uncertainty has driven interest in biomarkers that reflect oncogenic transformation and may improve diagnostic consistency.

The cyclin-dependent kinase inhibitor p16INK4a is overexpressed after HPV E7-mediated functional inactivation of the retinoblastoma pathway and is widely used as a surrogate marker of transforming high-risk HPV-associated disease [7]. Its expression has also been investigated as an indicator of progression risk in low-grade lesions [8].

Ki-67 reflects cellular proliferation and, when expressed beyond the basal or parabasal layers, indicates abnormal expansion of the proliferative compartment. p16/Ki-67 dual-staining strategies improve sensitivity for CIN2+ in HPV-positive screening populations [9,10]. Most available evidence, however, concerns screening triage rather than risk refinement after histologic abnormalities have already been identified.

The clinical question in a biopsy-confirmed referral population is therefore not whether biomarkers should determine referral to colposcopy but whether they contribute information beyond genotype and age when estimating the likelihood of clinically significant disease. The incremental value of p16 in this setting has seldom been quantified through nested predictive models, and the independent contribution of Ki-67 remains uncertain.

We therefore evaluated the diagnostic performance and incremental predictive contribution of p16 and Ki-67 in a consecutive real-world cervical biopsy cohort. We hypothesized that p16 would improve discrimination beyond age and HPV genotype, whereas Ki-67 would offer limited additional value after p16 was incorporated.

## 2. Materials and Methods

This retrospective cohort study was conducted in the Department of Gynecologic Oncology Surgery at İzmir City Hospital, Türkiye. The electronic pathology database was queried to identify patients who underwent index colposcopy-directed cervical biopsy between December 2023 and January 2025. Clinical, pathological, and follow-up records were reviewed through January 2026 to allow for completion of the prespecified 12-month surveillance period and ascertainment of final outcomes. Thus, January 2026 represented the end of the follow-up and outcome-ascertainment period rather than the date of the last index biopsy. Ethical approval was obtained from the Institutional Ethics Committee of İzmir City Hospital (approval no. 2026/118; 18 February 2026), and the study was conducted in accordance with the Declaration of Helsinki and institutional research standards.

All consecutive adults who underwent colposcopy-directed cervical biopsy between December 2023 and January 2025 were assessed for eligibility. Eligibility required high-risk HPV positivity; genotype information distinguishing HPV16, HPV18, and other high-risk types; interpretable p16 and Ki-67 immunohistochemistry on the index-biopsy specimen; and completed outcome ascertainment. Outcome ascertainment consisted of margin-negative excisional histopathology or, for women managed conservatively after a benign or LSIL biopsy, completion of 12-month surveillance with HPV testing and cytology. Conservatively managed patients were included in the final analytic cohort only if the required 12-month surveillance had been completed by the end of the outcome-ascertainment period; patients with incomplete surveillance were excluded rather than classified as disease-negative.

**Figure 1:**
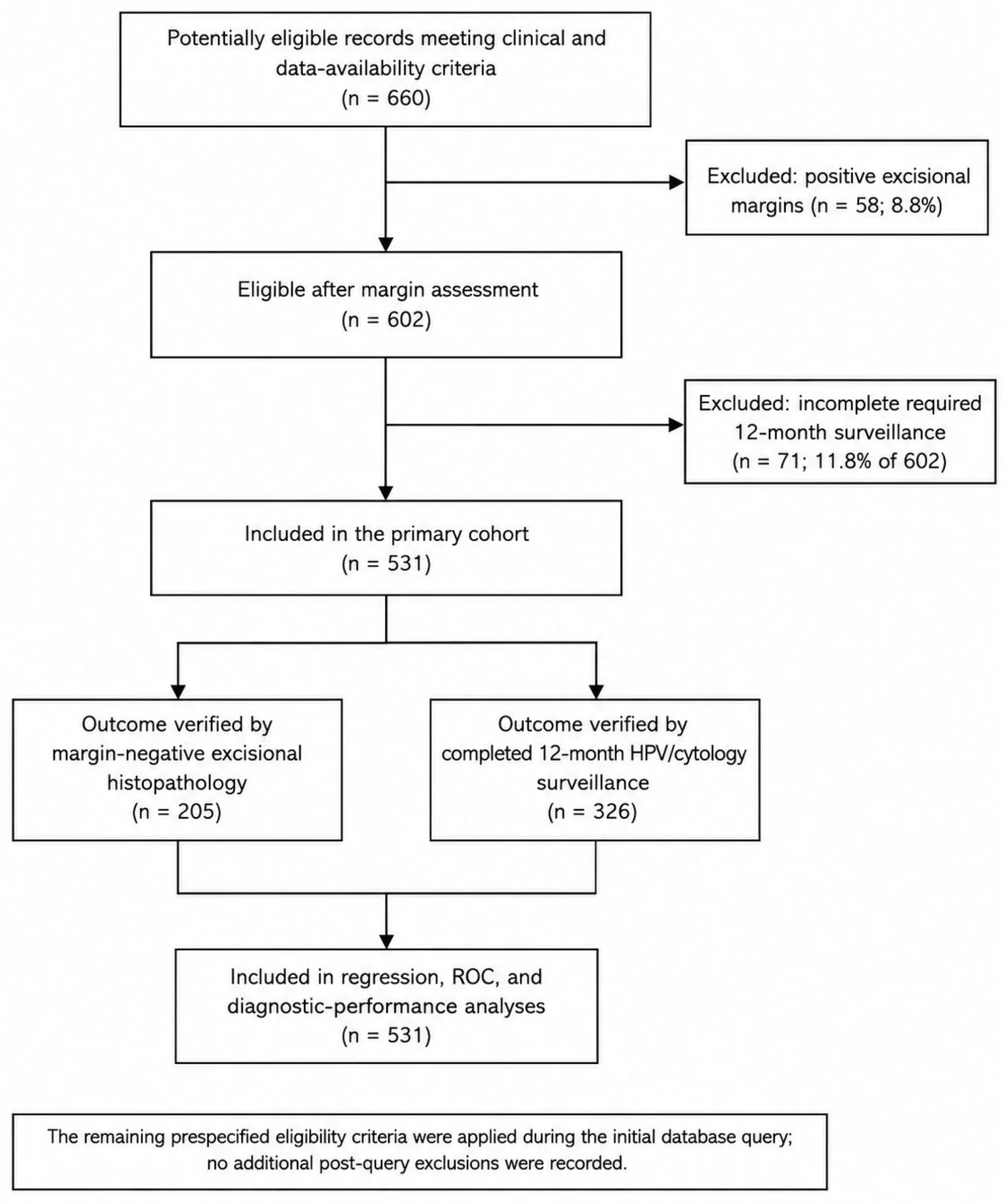
STROBE-style flow diagram of cohort selection and outcome ascertainment. Of 660 potentially eligible records, 58 with positive excisional margins were excluded. Among the 602 remaining patients, 71 did not complete the required 12-month surveillance; 531 were included. Outcomes were verified by margin-negative excisional histopathology in 205 and completed 12-month HPV/cytology surveillance in 326.

The database query identified 660 records meeting all prespecified clinical and data-availability criteria except the margin and follow-up requirements. Fifty-eight patients with positive excisional margins were excluded because residual disease could compromise biopsy-to-final-pathology correlation. Of the remaining 602 patients, 71 (11.8%) did not complete the required 12-month surveillance and were therefore excluded, leaving 531 patients in the final analytic cohort (Figure 1). Incomplete genotype or biomarker data, prior cervical excision, previous cervical malignancy, pregnancy, and inadequate biopsy material were screened during the initial database query; no additional post-query exclusions were recorded.

High-risk HPV testing and genotyping were performed with the Roche Cobas 6800 HPV Test (Roche Molecular Systems, Pleasanton, CA, USA), a validated real-time polymerase chain reaction assay. The platform reports HPV16 and HPV18 individually and pools 12 other oncogenic genotypes (31, 33, 35, 39, 45, 51, 52, 56, 58, 59, 66, and 68) into a single channel. Patients were therefore categorized as HPV16, HPV18, or other high-risk HPV. This grouping permitted evaluation of biomarker contribution beyond the two genotypes with the highest established risk, although it did not allow for type-specific assessment within the pooled category.

Colposcopy and directed biopsy were performed by experienced gynecologic oncology specialists. Loop electrosurgical excision or cold-knife conization was undertaken by the gynecologic oncology team according to standardized institutional protocols; the procedure was selected according to lesion characteristics, transformation-zone type, and clinical judgment.

All biopsy and excisional specimens were evaluated by one experienced gynecologic pathologist (D.T.), who was blinded to subsequent outcomes at the initial assessment. Diagnoses followed established cervical intraepithelial neoplasia criteria. CIN2+ comprised HSIL/CIN2-3, adenocarcinoma in situ (AIS), squamous cell carcinoma, and adenocarcinoma.

p16 and Ki-67 immunohistochemistry was performed on formalin-fixed, paraffin-embedded biopsy tissue using standardized laboratory procedures. p16 positivity required strong, continuous block-type nuclear and cytoplasmic staining. Ki-67 positivity required increased nuclear staining extending beyond the basal/parabasal layers, consistent with abnormal proliferative expansion. The same pathologist interpreted all stains to maintain within-study consistency.

Women with biopsy-confirmed HSIL, AIS, squamous cell carcinoma, or adenocarcinoma underwent excision. Women with benign histology or LSIL were managed conservatively and underwent repeat HPV testing and cytology at 12 months. Margin-negative excisional histopathology was the reference standard for treated patients. For conservatively managed patients, negative HPV testing together with benign cytology after the completed 12-month interval defined a negative final outcome; repeat biopsy was not routinely performed when both tests were negative.

The primary outcome was final CIN2+. Sensitivity, specificity, positive predictive value (PPV), and negative predictive value (NPV) were calculated with Wilson 95% confidence intervals (CIs), together with the underlying true-positive, false-positive, true-negative, and false-negative counts. Categorical comparisons used the chi-square or Fisher exact test, as appropriate. To examine the possible effect of differential outcome verification, a sensitivity analysis used the uniform index-biopsy diagnosis as the reference standard for all 531 patients.

Three nested logistic regression models were specified a priori on clinical and biological grounds rather than selected by univariable *p* values: model 1 included age and HPV genotype; model 2 added p16; and model 3 added Ki-67. Other high-risk HPV served as the genotype reference category. Adjusted odds ratios (aORs) with 95% CIs were reported. Multicollinearity was assessed using variance inflation factors (VIFs), with VIF < 5 considered acceptable. Discrimination was quantified by the area under the receiver operating characteristic curve (AUC), and nested model fit was compared using likelihood-ratio tests. Calibration and overall fit were evaluated using the Hosmer–Lemeshow test, Brier score, Nagelkerke R2, Akaike information criterion, and Bayesian information criterion.

**Figure 2:**
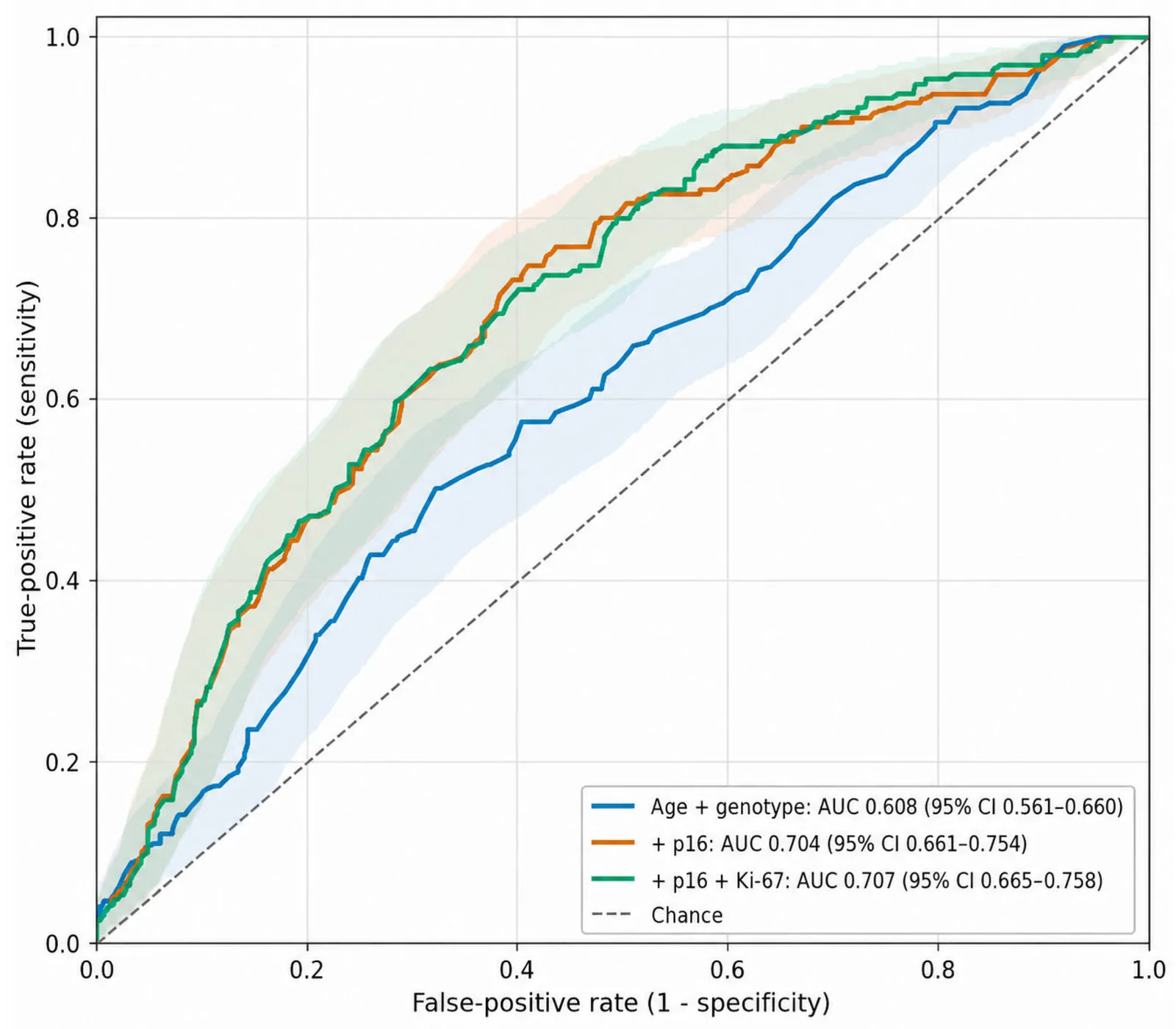
Receiver operating characteristic curves comparing predictive models for CIN2+. Shaded areas indicate 95% bootstrap confidence bands. AUCs and their bootstrap 95% confidence intervals are shown in the legend. Adding p16 improved discrimination beyond age and genotype, whereas Ki-67 produced minimal further gain. The shaded areas represent the 95% confidence bands for the corresponding ROC curves.

Internal validation used 2000 outcome-stratified bootstrap resamples. Apparent AUCs, percentile 95% CIs, optimism-corrected AUCs, and paired bootstrap CIs for ΔAUC were calculated. Primary analyses were conducted in IBM SPSS Statistics for Windows, version 28.0 (IBM Corp., Armonk, NY, USA); bootstrap validation and confidence bands for Figure 2 were implemented in Python 3.12 using SciPy (version 1.11.2) and scikit-learn (version 1.8.0). All tests were two-sided, and *p* < 0.05 was considered statistically significant.

## 3. Results

Figure 1 summarizes cohort selection and outcome ascertainment. The final analysis included 531 women with complete genotype, biomarker, and outcome data. Mean age was 41.3 ± 12.5 years; 251 (47.3%) had HPV16, 47 (8.9%) had HPV18, and 233 (43.9%) had other high-risk HPV. p16 and Ki-67 were positive in 321 (60.5%) and 418 (78.7%) women, respectively. CIN2+ was confirmed in 191 patients (36.0%) (Table 1).

### 3.1. Clinical Management Outcomes

Biopsy-guided management led to excision in 205 women (38.6%). Final excisional histopathology confirmed CIN2+ in 191, corresponding to a PPV of 93.2% for biopsy-indicated excision. Fourteen specimens (6.8%) were downgraded to ≤CIN1, and the number needed to excise to identify one CIN2+ lesion was 1.07.

The remaining 326 women were managed conservatively after benign or LSIL biopsy. All included women completed the prespecified 12-month interval and had negative HPV testing with benign cytology; no repeat biopsy was clinically indicated and no CIN2+ lesion was identified during surveillance.

### 3.2. Diagnostic Performance in the Overall Cohort

Table 2 presents diagnostic performance and the corresponding 2 × 2 counts. p16 identified 150 of 191 CIN2+ lesions (sensitivity 78.5%, 95% CI 72.2–83.8) and correctly classified 169 of 340 non-CIN2+ outcomes (specificity 49.7%, 95% CI 44.4–55.0). Its PPV was 46.7% (95% CI 41.3–52.2), and its NPV was 80.5% (95% CI 74.6–85.3).

Ki-67 identified 173 of 191 CIN2+ lesions (sensitivity 90.6%, 95% CI 85.6–94.0), but only 95 of 340 non-CIN2+ outcomes were correctly classified (specificity 27.9%, 95% CI 23.4–32.9). Its PPV was 41.4% (95% CI 36.8–46.2), and its NPV was 84.1% (95% CI 76.2–89.7).

Thus, the higher sensitivity of Ki-67 was achieved at the cost of substantially more false-positive results, whereas p16 provided a more balanced sensitivity-specificity profile.

### 3.3. Genotype-Stratified Distribution of CIN2+

CIN2+ prevalence differed by genotype: 102/251 (40.6%) for HPV16, 22/47 (46.8%) for HPV18, and 67/233 (28.8%) for other high-risk HPV. The high point estimate in the small HPV18 subgroup should be interpreted cautiously.

### 3.4. Genotype-Stratified Biomarker Performance

Table 3 presents genotype-stratified performance, including Wilson 95% CIs for all predictive values and the corresponding cell counts. Among HPV16-positive women, p16 sensitivity and specificity were 82.4% (95% CI 73.8–88.5) and 53.7% (95% CI 45.7–61.5), respectively; PPV and NPV were 54.9% (95% CI 47.0–62.6) and 81.6% (95% CI 72.8–88.1). Ki-67 sensitivity was 93.1% (95% CI 86.5–96.6), but specificity was 27.5% (95% CI 21.0–35.2); PPV and NPV were 46.8% (95% CI 40.1–53.7) and 85.4% (95% CI 72.8–92.8).

In the HPV18 subgroup, p16 sensitivity was 90.9% (95% CI 72.2–97.5) and specificity was 48.0% (95% CI 30.0–66.5); PPV and NPV were 60.6% (95% CI 43.7–75.3) and 85.7% (95% CI 60.1–96.0). Ki-67 identified all 22 CIN2+ cases (sensitivity 100%, 95% CI 85.1–100), with specificity 36.0% (95% CI 20.2–55.5), PPV 57.9% (95% CI 42.2–72.1), and NPV 100% (95% CI 70.1–100). These wide intervals, particularly for specificity and NPV, reflect the limited subgroup size (*n* = 47) and preclude firm genotype-specific conclusions.

For other high-risk HPV, p16 sensitivity was 68.7% (95% CI 56.8–78.5), specificity 46.4% (95% CI 39.0–54.0), PPV 34.1% (95% CI 26.6–42.4), and NPV 78.6% (95% CI 69.5–85.5). Corresponding Ki-67 estimates were 83.6% (95% CI 72.9–90.6), 27.1% (95% CI 20.9–34.3), 31.6% (95% CI 25.2–38.8), and 80.4% (95% CI 68.2–88.7).

Both biomarkers were more sensitive in HPV16- and HPV18-positive disease than in the pooled other-high-risk group, while specificity remained modest in every genotype category.

### 3.5. Multivariable Analysis

The fully adjusted model is shown in Table 4. Relative to other high-risk HPV, HPV16 independently predicted CIN2+ (aOR 1.72, 95% CI 1.15–2.57; *p* = 0.0077). HPV18 had a similar point estimate but a wider CI that included the null (aOR 1.91, 95% CI 0.97–3.77; *p* = 0.0608).

Each additional year of age was associated with a 2.7% increase in the odds of CIN2+ (aOR 1.027, 95% CI 1.011–1.043; *p* = 0.0008).

p16 remained independently associated with CIN2+ (aOR 2.97, 95% CI 1.81–4.89; *p* <0.001). Ki-67 did not retain an independent association after p16 and the other covariates were included (aOR 1.74, 95% CI 0.90–3.35; *p* = 0.098).

### 3.6. Model Discrimination and Incremental Value

Figure 2 and Table 5 summarize model discrimination and internal validation. The age-plus-genotype model had an apparent AUC of 0.608 (bootstrap 95% CI 0.561–0.660). Adding p16 increased the AUC to 0.704 (ΔAUC 0.097, 95% CI 0.057–0.138); model fit also improved (likelihood-ratio χ^2^ = 43.97, 1 df; *p* <0.001). The corresponding optimism-corrected AUCs were 0.599 and 0.696.

Adding Ki-67 produced an apparent AUC of 0.707 (bootstrap 95% CI 0.665–0.758), representing only a 0.003 increase (95% CI −0.002 to 0.015). The likelihood-ratio test was not significant (χ^2^ = 2.79, 1 df; *p* = 0.095), and the optimism-corrected AUC was 0.697.

VIFs ranged from 1.01 to 1.53, excluding meaningful multicollinearity. The full model showed acceptable calibration (Hosmer–Lemeshow *p* = 0.645), a Brier score of 0.203, and a Nagelkerke R2 of 0.161. AIC/BIC values were 682.37/699.47 for model 1, 640.40/661.77 for model 2, and 639.60/665.25 for model 3. When the uniform index-biopsy diagnosis was used as the outcome for all 531 women, AUCs were 0.610, 0.723, and 0.725 for models 1–3, respectively. This sensitivity analysis reproduced the primary pattern: a substantial increment with p16 and negligible additional discrimination with Ki-67.

### 3.7. Counterfactual Biomarker-Based Management Strategies

If excision had been restricted to p16-positive women, 321 procedures would have been performed and 41 CIN2+ cases would have been missed. Requiring both p16 and Ki-67 positivity would have resulted in 315 procedures and 43 missed CIN2+ cases. Both counterfactual strategies increased the procedural burden relative to observed biopsy-guided care while introducing clinically important false-negative classifications.

## 4. Discussion

In this referral cohort, age and HPV genotype alone provided limited discrimination for CIN2+. p16 supplied a clear and internally validated increment, whereas Ki-67 contributed little after p16 was known. The uniform biopsy-reference sensitivity analysis produced the same ranking of models, supporting the robustness of the central finding to the outcome-verification method.

The progressive increase in p16 sensitivity from other high-risk HPV to HPV16 and HPV18 is biologically plausible because block-type p16 expression reflects disruption of the pRb pathway and transforming viral activity rather than viral detection alone [11,12]. This interpretation is consistent with the role assigned to p16 in the LAST framework and with broader efforts to improve reproducibility in cervical lesion classification [13,14].

The present analysis extends these observations by quantifying the information gained beyond genotype and age. The association between multiple high-risk infections and p16 expression reported by Zhang et al. similarly suggests that p16 captures the biological effect of oncogenic exposure that cannot be represented fully by a pooled genotype category [15].

The clinical meaning of p16 depends on setting. De Sam Lazaro et al. proposed that p16-guided assessment might reduce unnecessary excision in selected biopsy populations [16]. In our cohort, however, biopsy-indicated excision already had a PPV of 93.2%, and p16-only management would have increased procedures while missing 41 CIN2+ lesions. Accordingly, p16 is better interpreted here as a risk modifier than as an alternative treatment threshold.

This context-specific interpretation accords with management-focused evidence from Ebisch et al. and population-screening findings from Sardinha et al., both of which linked p16 positivity to CIN2+ while emphasizing its adjunctive role [17,18]. It also explains why results from screening studies of p16/Ki-67 dual staining, including IMPACT [10], cannot be transferred directly to a histologically enriched referral cohort.

Ki-67 achieved high sensitivity but very low specificity, generating 245 false-positive results in the overall cohort. A binary definition based on suprabasal extension may favor sensitivity at the expense of specificity. Future studies should compare prespecified quantitative indices, zonal distribution, staining intensity, or combined architectural patterns and should evaluate whether such refinements improve discrimination without sacrificing reproducibility.

Avoiding unnecessary excision remains important because treatment-related obstetric risk increases with excision depth [19]. Nevertheless, the present counterfactual analysis shows that reducing treatment solely through biomarker thresholds would exchange overtreatment for missed high-grade disease. Biomarker information should therefore be integrated with morphology and clinical risk rather than used in isolation.

Several sources of uncertainty warrant emphasis. The dual reference standard could miss occult CIN2+ in conservatively managed women because negative 12-month cotesting does not provide the same tissue verification as excision. Although the uniform biopsy-reference analysis was reassuring, it cannot eliminate this concern. The HPV18 estimates were based on only 47 women and consequently had wide CIs. In addition, Cobas 4800 pooled 12 high-risk genotypes; clinically relevant heterogeneity among HPV31, 33, 52, and 58 could therefore not be examined. The occurrence of HPV-negative CIN2+ in ATHENA further illustrates why genotype categories alone cannot capture all biologically significant disease [20].

The tertiary gynecologic oncology setting enriched CIN2+ prevalence and limits direct transportability to community colposcopy practice. Because predictive values vary with prevalence, p16 PPV would probably be lower in a standard clinic, although its adjusted association may remain informative. External validation and recalibration in lower-prevalence populations are required before the model can support clinical thresholds.

Other limitations include the retrospective single-center design, exclusion of 71 women with incomplete surveillance, and exclusion of 58 margin-positive cases, each of which may introduce selection bias. The excluded margin-positive cases could not be characterized comparatively because patient-level variables were not retained in the analytic dataset. All histology and immunohistochemistry were interpreted by one pathologist. This reduced within-study variation but prevented assessment of interobserver reproducibility, an important consideration for p16-assisted classification. Finally, bootstrap correction addresses optimism but does not substitute for external validation.

Strengths include consecutive real-world sampling, explicit genotype stratification, transparent 2 × 2 reporting, prespecified nested models, calibration assessment, bootstrap internal validation, and a uniform-reference sensitivity analysis. Taken together, the data support p16 as an adjunct for genotype-informed risk refinement while providing no evidence that Ki-67 materially improves prediction once p16 is available.

## 5. Conclusions

In this real-world cervical biopsy cohort, p16 improved prediction of CIN2+ beyond age and HPV genotype, and the gain persisted after bootstrap correction and under a uniform biopsy-reference sensitivity analysis. Ki-67 was highly sensitive but insufficiently specific and did not provide a statistically reliable increment after p16 was incorporated.

These biomarkers should not replace histopathology-guided management. p16 may instead serve as an adjunctive risk modifier, provided that its use is externally validated and recalibrated before application in lower-prevalence community settings.

## Figures and Tables

**Table 1:** Baseline Characteristics, Biomarker Distribution, and Outcomes.

Characteristic	Total (*n* = 531)
Age, years, mean ± SD	41.3 ± 12.5
**HPV genotype**	
HPV16	251 (47.3%)
HPV18	47 (8.9%)
Other high-risk HPV	233 (43.9%)
p16 positive	321 (60.5%)
Ki-67 positive	418 (78.7%)
**Index-biopsy category**	
Benign/negative	186 (35.0%)
LSIL/CIN1	140 (26.4%)
HSIL/CIN2+	159 (29.9%)
AIS	32 (6.0%)
Squamous cell carcinoma	9 (1.7%)
Adenocarcinoma	5 (0.9%)
Final CIN2+	191 (36.0%)
Final ≤ CIN1	340 (64.0%)
Excision performed	205 (38.6%)
CIN2+ among excised patients	191/205 (93.2%)
Downgrade to ≤CIN1 after excision	14/205 (6.8%)

**Table 2:** Diagnostic Performance of p16 and Ki-67 for CIN2+ in the Overall Cohort.

Marker	TP/FP/TN/FN	Sensitivity, % (95% CI)	Specificity, % (95% CI)	PPV, % (95% CI)	NPV, % (95% CI)
p16	150/171/169/41	78.5 (72.2–83.8)	49.7 (44.4–55.0)	46.7 (41.3–52.2)	80.5 (74.6–85.3)
Ki-67	173/245/95/18	90.6 (85.6–94.0)	27.9 (23.4–32.9)	41.4 (36.8–46.2)	84.1 (76.2–89.7)

**Table 3:** Genotype-Stratified Diagnostic Performance.

**HPV16 (*n* = 251)**					
**Marker**	**TP/FP/TN/FN**	**Sensitivity, % (95% CI)**	**Specificity, % (95% CI)**	**PPV, % (95% CI)**	**NPV, % (95% CI)**
p16	84/69/80/18	82.4 (73.8–88.5)	53.7 (45.7–61.5)	54.9 (47.0–62.6)	81.6 (72.8–88.1)
Ki-67	95/108/41/7	93.1 (86.5–96.6)	27.5 (21.0–35.2)	46.8 (40.1–53.7)	85.4 (72.8–92.8)
**HPV18 (*n* = 47)**					
**Marker**	**TP/FP/TN/FN**	**Sensitivity, % (95% CI)**	**Specificity, % (95% CI)**	**PPV, % (95% CI)**	**NPV, % (95% CI)**
p16	20/13/12/2	90.9 (72.2–97.5)	48.0 (30.0–66.5)	60.6 (43.7–75.3)	85.7 (60.1–96.0)
Ki-67	22/16/9/0	100 (85.1–100)	36.0 (20.2–55.5)	57.9 (42.2–72.1)	100 (70.1–100)
**Other high-risk HPV (*n* = 233)**					
**Marker**	**TP/FP/TN/FN**	**Sensitivity, % (95% CI)**	**Specificity, % (95% CI)**	**PPV, % (95% CI)**	**NPV, % (95% CI)**
p16	46/89/77/21	68.7 (56.8–78.5)	46.4 (39.0–54.0)	34.1 (26.6–42.4)	78.6 (69.5–85.5)
Ki-67	56/121/45/11	83.6 (72.9–90.6)	27.1 (20.9–34.3)	31.6 (25.2–38.8)	80.4 (68.2–88.7)

**Table 4:** Multivariable Logistic Regression for CIN2+.

Variable	Adjusted OR	95% CI	*p* Value
HPV16 vs. other high-risk HPV	1.72	1.15–2.57	0.0077
HPV18 vs. other high-risk HPV	1.91	0.97–3.77	0.0608
Age, per year	1.027	1.011–1.043	0.0008
p16 positive	2.97	1.81–4.89	<0.001
Ki-67 positive	1.74	0.90–3.35	0.098

**Table 5:** Internal Validation and Uniform-Reference Sensitivity Analysis.

Reference Outcome	Model	Apparent AUC (95% Bootstrap CI)	Optimism-Corrected AUC	ΔAUC (95% Bootstrap CI)
Primary dual reference	Age + genotype	0.608 (0.561–0.660)	0.599	Reference
Primary dual reference	+p16	0.704 (0.661–0.754)	0.696	0.097 (0.057–0.138)
Primary dual reference	+p16 + Ki-67	0.707 (0.665–0.758)	0.697	0.003 (−0.002 to 0.015)
Uniform index biopsy	Age + genotype	0.610	-	Reference
Uniform index biopsy	+p16	0.723	-	0.114
Uniform index biopsy	+p16 + Ki-67	0.725	-	0.002

## Data Availability

The datasets generated and/or analyzed during the current study are available in the Zenodo repository: https://doi.org/10.5281/zenodo.18841837.

## Abbreviations

HPV, human papillomavirus; CIN, cervical intraepithelial neoplasia; HSIL, high-grade squamous intraepithelial lesion; AIS, adenocarcinoma in situ; PPV, positive predictive value; NPV, negative predictive value; ROC, receiver operating characteristic; AUC, area under the curve; aOR, adjusted odds ratio; CI, confidence interval; VIF, variance inflation factor; LEEP, loop electrosurgical excision procedure.
